# Severe asthma and allergy - should we look for the allergens

**DOI:** 10.1186/2045-7022-5-S2-P16

**Published:** 2015-03-23

**Authors:** Snezhina Lazova, Guergana Petrova, Dimitrinka Miteva, Vera Papochieva, Ljupco Zafirovski, Penka Perenovska

**Affiliations:** 1 UH "Alexandrovska", Pediatric clinic, Sofia, Bulgaria; 2 Children’s Hospital for Respiratory Diseases and TB, Respiratory departement, Skopje, Macedonia

## Background

Severe asthma is a heterogeneous condition consisting of phenotypes such as eosinophilic asthma. Usually atopy presence or any allergic diseases could interfere and obstacle the control of asthma. Even specific recommendations on the use of sputum eosinophil count and exhaled nitric oxide to guide therapy, as well as treatment with anti-IgE antibody are provided.

## Material and method

For a period of 6 months we evaluated medical history data of 29 children with asthma divided into two groups - 14 (6 girls, 8 boys) with severe asthma (SA) on high dose combined inhaled corticosteroids (ICS) and 15 (6 girls, 9 boys) with moderate asthma, matched by age to serve as a control group. For all children we performed - pulmonary function tests (PFT), nasal smears for eosinophil counts, drew blood for IgE against inhalation and food allergies antibodies detection and ACQ. IgE were detected with the predesigned kit - EurolinePediatric.

## Results

The children with SA showed tendency for lower scores in ACQ and PFT without statistical significance (p>0,05). All children with SA had concomitant allergic rhinitis on contrast with the control group, where only 3(20%) had also allergic rhinitis. The nasal smears had higher eosinophil levels in SA group (p<0,05). 92,85%(13 children) from SA group and 33.33% (5 children) had minimum 1 elevated antigen titer (at least 0.7 kU/l). High and very high antibody titer (with clinical significance) was noted in 11 children with SA and 2 in control group. The most common allergens in SA group were (with clinical significance) was noted in 11 children with SA and 2 in control group. The most common clinically significant allergens in SA group were dermatophagoides (7 patients), grass mix (5 patients) and cat (4 patients), alternaria and birch were also found in 3 and 2 patients respectively. For control group 1 patient had high titer against cat and the other against grass mix.

## Conclusion

Precise detection of the allergen could help not only in maintaining better control in SA patients with avoidance technique, but also in designing a recommendation plan in patients who are unable to be tested.

